# Critical residues involved in Toll-like receptor 4 activation by cationic lipid nanocarriers are not located at the lipopolysaccharide-binding interface

**DOI:** 10.1007/s00018-015-1915-1

**Published:** 2015-05-09

**Authors:** Caroline Lonez, Kate L. Irvine, Malvina Pizzuto, Boris I. Schmidt, Nick J. Gay, Jean-Marie Ruysschaert, Monique Gangloff, Clare E. Bryant

**Affiliations:** 1 Department of Veterinary Medicine, University of Cambridge, Cambridge, UK; 2 Structure and Function of Biological Membranes, Université Libre de Bruxelles, Brussels, Belgium; 3 Department of Biochemistry, University of Cambridge, Cambridge, UK

**Keywords:** Toll-like receptor, Cationic lipid, Nanoparticle, Activation mechanism, Species specificity

## Abstract

DiC14-amidine is a cationic lipid that was originally designed as a lipid nanocarrier for nucleic acid transport, and turned out to be a Toll-like receptor 4 (TLR4) agonist as well. We found that while *E. coli* lipopolysaccharide (LPS) is a TLR4 agonist in all species, diC14-amidine nanoliposomes are full agonists for human, mouse and cat receptors but weak horse agonists. Taking advantage of this unusual species specificity, we used chimeric constructs based on the human and horse sequences and identified two regions in the human TLR4 that modulate the agonist activity of diC14-amidine. Interestingly, these regions lie outside the known LPS-binding domain. Competition experiments also support our hypothesis that diC14-amidine interacts primarily with TLR4 hydrophobic crevices located at the edges of the TLR4/TLR4* dimerization interface. We have characterized potential binding modes using molecular docking analysis and suggest that diC14-amidine nanoliposomes activate TLR4 by facilitating its dimerization in a process that is myeloid differentiation 2 (MD-2)-dependent and cluster of differentiation 14 (CD14)-independent. Our data suggest that TLR4 may be activated through binding at different anchoring points, expanding the repertoire of TLR4 ligands to non-MD-2-binding lipids.

**Electronic supplementary material:**

The online version of this article (doi:10.1007/s00018-015-1915-1) contains supplementary material, which is available to authorized users.

## Introduction

Toll-like receptor 4 (TLR4) is a member of the innate immune system’s Pattern Recognition Receptor (PRR) family, specialized in the recognition of bacterial lipopolysaccharides (LPS), components of the outer membrane of Gram-negative bacteria. The interaction between TLR4 and LPS requires two main co-receptors: the Myeloid Differentiation Factor 2 (MD-2), a glycoprotein physically associated with TLR4 on the cell surface and conferring to TLR4 its responsiveness to LPS and the Cluster of Differentiation 14 (CD14) which is believed to transport the LPS into the vicinity of TLR4/MD-2. Upon agonist recognition, TLR4/MD-2 homodimerizes and activates two main signalling pathways that depend on the adaptors recruited, both triggering the production of pro-inflammatory cytokines and chemokines: the Myeloid Differentiation primary response 88 (MyD88)-dependent cascade leading to the activation of the Nuclear Factor kappa B (NF-κB) and Activated Protein-1 (AP-1) transcription factors and the TIR-domain-containing adapter-inducing interferon-β (TRIF)-dependent pathway leading to the activation of the Interferon Regulatory Factor 3 (IRF-3) [1, 2].

The resolved structure of the dimeric complex TLR4/MD-2 with bound LPS [3] has revealed that 5 acyl chains of the hexa-acylated *Escherichia coli* LPS (abbrv. *E. coli* LPS or EC-LPS) are buried deep inside a hydrophobic pocket in MD-2, with the 6th acyl chain partially exposed to the surface of the protein, participating in the dimerization interface. In contrast, lipid IVa, a tetra-acylated precursor of *E. coli* LPS, which is an antagonist in human, is completely buried inside the hydrophobic pocket of MD-2 in a conformation that prevents TLR4 dimerization [4]. Depending on their structure (i.e. acylation pattern, number of phosphate groups), but also on the TLR4 and MD-2 mammalian species, LPS from different natural origins or synthetic LPS derivatives will bind and induce or prevent signalling with different efficiencies [5–8]. Penta-acylated lipopolysaccharide from *Rhodobacter sphaeroides* (RS-LPS) acts as agonists of TLR4 in horses and hamsters, but as an antagonist in humans and mice [9–13]; lipid IVa, mentioned earlier, acts as an antagonist in human, but as an agonist in mouse, hamster, horse and cow [5, 9, 14–17]. Therefore, swapping experiments in which TLR4 from one species is used in combination with MD-2 from another species and inter-species chimera where amino acids found in one species are mutated with the corresponding residues from another species have allowed to identify regions in both TLR4 and MD-2 involved in species dependency of TLR4 ligands [14, 16, 18–20]. Recently, the comparison of the crystal structures of mouse TLR4/MD-2/lipid IVa (agonist) [17] and human MD-2/lipid IVa (antagonist) [4] confirmed the data obtained using species dependency and revealed that specific residues present in both mTLR4 and mMD-2 modulate the charge distribution of the complex, favouring the agonist positioning of lipid IVa in mouse TLR4 which promotes dimerization of mTLR4/MD-2/lipid IVa [17].

We showed previously that a cationic lipid synthesized in our laboratory, diC14-amidine [21, 22] (Fig. S1), activates TLR4 and MD-2-dependent MyD88 and TRIF-dependent signalling pathways in human and murine dendritic cells [23–26]. DiC14-amidine’s structure differs noticeably from the LPS structure (Fig. S1) both in size and charge, and aggregates into liposomes. We therefore hypothesized that the interaction mode of diC14-amidine nanoliposomes with TLR4/MD-2 would be different from traditional ligands such as LPS.

## Materials and methods

### Constructs

pcDNA3-hTLR4, pcDNA3-eTLR4, pcDNA3-hCD14, pEFIRES-hMD-2 and pEFIRES-eMD-2 were constructed as described earlier [16]. TLR4 chimeras were constructed by overlap extension PCR and point mutations were introduced by site-directed mutagenesis (QuickChange; Stratagene) and mutations were confirmed by sequencing as described [16]. The ten different chimeras we used in this work are represented in Fig. S3: the first group corresponds to human TLR4 (H) in which a specific region has been replaced by its corresponding equine insert (E) to generate HE chimeras; the second group possesses the equine backbone (E) which is locally replaced by human regions (EH chimeras). Constructs that failed to signal to LPS were excluded from this study.

### Materials

DiC14-amidine was synthesized as described earlier [21] and stored as powder at −20 °C. Lipid films were formed by dissolving powder in chloroform, followed by solvent evaporation under nitrogen stream, vacuum drying overnight, and storage at −20 °C. Before each experiment, lipid films were freshly resuspended in filtered Hepes 10 mM heated at 55 °C as previously described [22].

EC-LPS (UltraPure LPS—*Escherichia coli* O111:B4 subtype) and RS-LPS (*Rhodobacter sphaeroides LPS*) were obtained from InvivoGen and were freshly prepared for each experiment in water at a concentration of 1 mg/ml by vortexing, followed by sonication for 1 min. LPS at 100 ng/ml corresponds approximately to 5–15 nM.

All cell culture media and components were purchased at Lonza.

### Cell culture and transient transfection

Human embryonic kidney cells (HEK 293) were obtained from ATCC (Manassas, VA, USA). Cells were maintained in DMEM supplemented with 10 % FCS, 2 mM l-glutamine, 100 U/ml penicillin and 100 μg/ml streptomycin. HEK293 cells were transfected as previously described [16]. Briefly, cells were seeded at 7.5 × 10^4^ cells/well in a 96-well plate and transiently transfected 3 days later. Expression vectors containing cDNA encoding TLR4 (5 ng/well), MD-2 (1 ng/well), and CD14 (1 ng/well), a NF-κB transcription reporter vector encoding *Firefly* luciferase (10 ng/well pNF-κB-luc; Clontech) and a constitutively active reporter vector encoding *Renilla* luciferase (5 ng/well phRG-TK; Promega) together with empty vector ensure that an optimal amount of DNA was mixed with jetPEI (Polyplus transfection) according to the manufacturer’s instructions.

After 48 h cells were stimulated for 6 h with diC14-amidine (in serum-free DMEM), UltraPure LPS in complete medium, or NiCl_2_ (Sigma Aldrich) in complete medium. Cells were then washed with PBS and lysed with Passive Lysis Buffer (Promega). *Luciferase* and *Renilla* activity were then quantified on a FLUOstar Omega (BMG Labtech) using home-made luciferase reagent [20 mM Tricine, 2.67 mM MgSO_4_.7H_2_O, 0.265 mM (MgCO_3_)_4_Mg(OH)_2_.5H_2_O, 0.1 mM EDTA, 33.3 mM DTT, 530 μM ATP, 270 μM Acetyl CoEnzyme A (Lithium salt), 470 µM Luciferin (Biosynth), pH 7.8, diluted 2 times in water before use] or coelenterazine (Biosynth) dissolved in ethanol at 1 mg/ml and diluted 500 times in PBS before use as described in [16]. Luciferase luminescence intensity was normalized to renilla luminescence intensity and data were expressed as fold induction as compared to non-induced control or as percentage as compared to LPS. Renilla luminescence serves as a control for experiment-inherent minor variations concerning cell numbers and transfection efficiencies between individual wells on the used microtiter plates. All transfected cells were tested for their ability to respond to EC-LPS in parallel to other ligands to ensure that the MD-2/TLR4 constructs were functional and to control any differences in protein expression efficiency.

For competition assays, 48 h after transfection, cells were incubated with RS-LPS for 1 h, then cells were washed and stimulated as described. We used cells pretreated with RS-LPS before diC14-amidine stimulation or EC-LPS stimulation rather than co-administration, to prevent direct contact between lipids of opposing charges which could lead to possible interference within their respective micellar structures rather than at the level of receptor binding.

The THP1 cells were obtained from ATCC (Manassas, VA, USA) and were maintained in RPMI medium with 25 mM HEPES supplemented with 10 % FBS, 2 mM l-glutamine, 100 U/ml penicillin and 100 μg/ml streptomycin, 1 mM sodium pyruvate and 20 µM 2-Mercaptoethanol, at 37 °C at 5 % CO_2_.

For experiments, cells were primed with 10 nM phorbol 12-myristate 13-acetate (PMA—Sigma Aldrich) to induce differentiation 28 h before stimulation. After this incubation period, the PMA-containing medium was removed and replaced with PMA-free complete medium for 4 h at 37 °C at 5 % CO_2_. Cells were then preincubated with the indicated amounts of neutralizing antibodies against human CD14 (InvivoGen) or Control antibody (InvivoGen) at a final concentration of 20 µg/mL for 1 h, then stimulated with the indicated amounts of EC-LPS or diC14-amidine in serum-free medium (added in concentrated form into the antibody-containing medium to reach their final stimulant concentrations). After 4 h incubation, the supernatants were recovered and analysed by ELISA following manufacturer’s instructions (DuoSet kits from R&D Systems).

### Statistical analysis

Multiple comparisons versus control group for each treatment within groups were made using One-Way ANOVA (Holm–Sidak method) or Kruskal–Wallis One-Way Analysis of Variance on Ranks (Dunnett’s method) when normality test failed, using SigmaPlot software.

### Computational methods

#### DiC14-amidine model

The molecular structure of diC14-amidine was generated ab initio in Sybyl software version 8.1.1 (Tripos). The geometry of the lipid was optimized using the Powell minimisation method, with initial optimization based on the Simplex method, and with a gradient of 0.05 kcal/mol and a maximum of 100 cycles of iteration. Partial charges were computed based on the Gasteiger–Hückel charge method.

#### TLR4 and MD-2 templates

The molecular structures of the extracellular region of human TLR4 on its own and bound to MD-2 proteins as observed in the crystal structure of the LPS complex [3] were used for surface visualization and molecular docking of diC14-amidine molecules. Docking experiments were performed upon removal of the *E. coli* LPS ligands from the coordinate file.

#### Molecular docking

Autodock Vina software package [27] was used for docking diC14-amidine on TLR4-MD-2. The TLR4:MD-2 dimeric receptor complex was treated as a rigid protein complex. DiC14-amidine was fully flexible as its 30 torsion angles are within the maximum allowed limit. The Autogrid parameters were computed for the entire TLR4-MD-2 complex, with a grid sized 100 × 100 × 100 Å^3^, but also for smaller areas centred on the regions shown to be important by mutagenesis, with a grid size of 40 × 40 × 40 Å^3^. The grid was centred on the complex at *x* = + 12.322; *y* = −7.964; *z* = −5.891. The default optimization parameters for the iterated local search global optimizer of Vina were used except for exhaustiveness, which was increased proportionally to the size of the grid (the default value of 8 was increased up to 32). Docking poses of the ligand were analysed and structural images were generated in PyMol (http://www.pymol.org), Chimera [28], and LigPlot [29].

## Results

### CD14 is not required in diC14-amidine activity

**Fig. 1:**
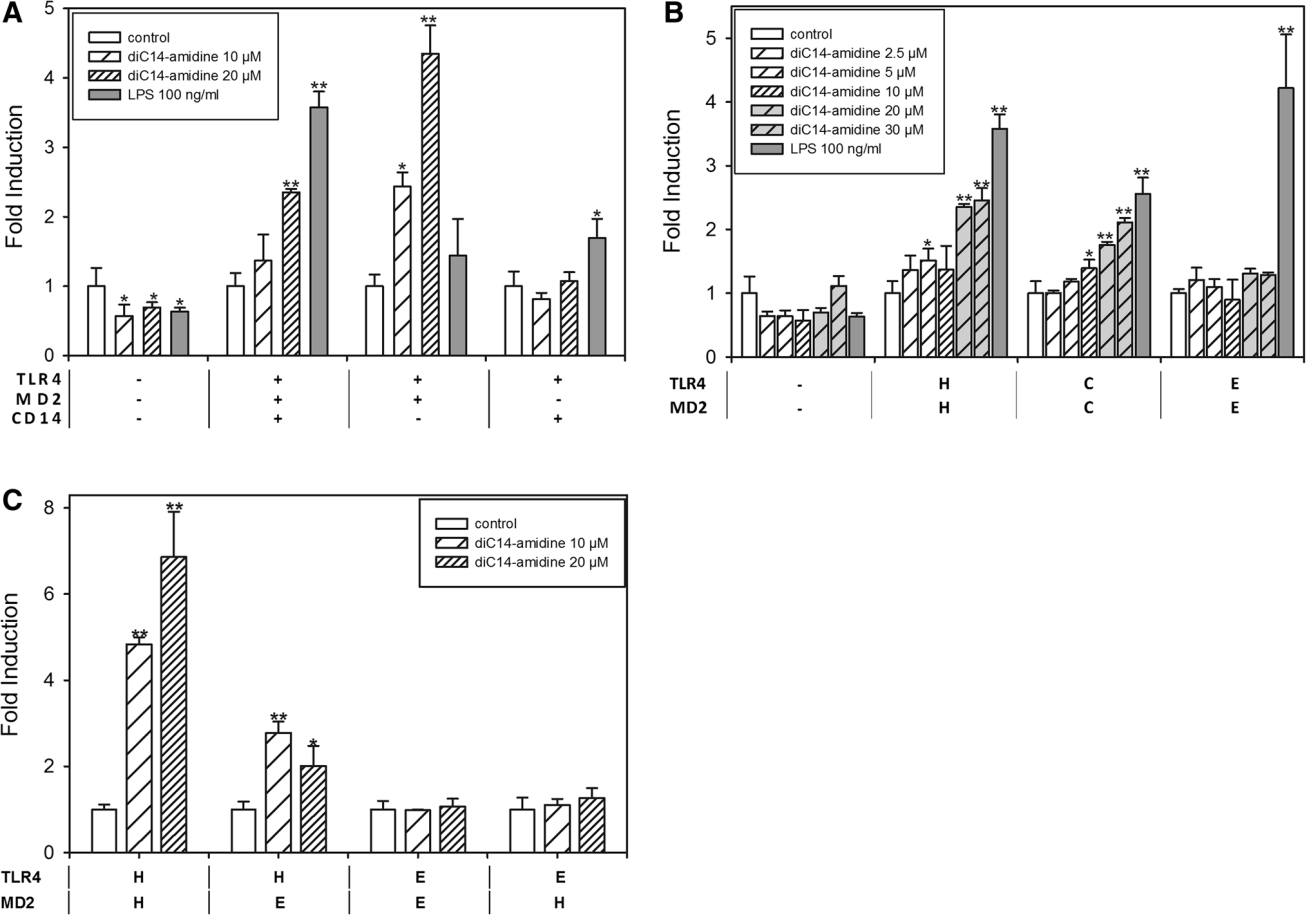
Human TLR4 drives the activity of diC14-amidine. HEK 293 cells were transfected with plasmids encoding human TLR4 and/or MD-2 and/or CD14 (**a**) or TLR4 and MD2 from different species (**b**, **c**) with (**b**) or without (**c**) human CD14 together with firefly luciferase reporter plasmid dependent of NF-κB activation. Two days after transfection, cells were stimulated for 6 h with diC14-amidine or LPS. Luciferase was then quantified in cell lysates. Data are represented as fold induction as compared to non-stimulated control for each condition. Means are expressed ± standard deviation with *n* = 3. Representative of at least 2 independent experiments. *p < 0.05, **p < 0.01 as compared to control (ANOVA). **a** Activation of NF-κB by diC14-amidine requires both TLR4 and MD-2 but not CD14. **b** Species-dependent activity of diC14-amidine. *H* human, *C* cat, *E* equine. **c** Partial activation of NF-κB by diC14-amidine is maintained in the presence of human TLR4

To decipher the TLR4 activation mechanism induced by diC14-amidine, we wanted in a first step to determine the role of the co-receptors MD-2 and CD14 in the agonist activity of diC14-amidine. The importance of TLR4 and MD-2 was already demonstrated by the inability of TLR4^−/−^ and MD-2^−/−^ bone marrow-derived dendritic cells to secrete IL-12p40 in response to diC14-amidine stimulation; however, the role of CD14 was not fully addressed so far [23]. We therefore transfected HEK293 cells with the plasmids coding for each protein in different combinations (Fig. 1a) and the cells were then stimulated with diC14-amidine liposomes or EC-LPS in the absence of serum.

As expected, in the absence of TLR4, MD-2 and CD14 no activation was observed for both ligands. Further, neither EC-LPS nor diC14-amidine was able to activate NF-κB in the absence of MD-2 even in the presence of TLR4 and CD14 (Fig. 1a, right). However, while EC-LPS is unable to activate TLR4/MD-2 in the absence of CD14, diC14-amidine’s TLR4 agonist activity was not affected by the lack of CD14, and was even found to have slightly increased.

We then evaluated CD14 requirement for NF-κB but also for IRF-3 induction on THP1 cells (by quantifying TNF-α and IP-10 in supernatants) by inhibiting CD14 using blocking antibodies (Fig. S2). This clearly confirms the non-requirement of CD14 for NF-κB activation by diC14-amidine, but more surprisingly, stimulation of the cells with diC14-amidine also resulted in the secretion of hIP10 when CD14 was neutralized, demonstrating that the cationic lipid also does not require CD14 to trigger the TRIF-dependent pathway.

### Species-specific activity of diC14-amidine

We previously showed that diC14-amidine is a TLR4 agonist in both human and mouse dendritic cells [23]. To understand the way diC14-amidine interacts with TLR4/MD-2, we compared the TLR4 agonist activity of diC14-amidine in two further species (cat and horse) by transfecting HEK293 cells with plasmids coding for TLR4, MD2 and CD14 from human (H), cat (C) and horse (E). We demonstrated (Fig. 1b) that, while EC-LPS is an agonist in all species, diC14-amidine is a full agonist for human and cat receptors, but induced low levels of activation in horse. Since we were unable to detect an antagonist effect of diC14-amidine on the full agonist EC-LPS (*data not shown*), we therefore consider this compound to be a weak agonist in horse TLR4. This suggests that diC14-amidine is unable to induce signal transduction in the horse, while it binds and activates TLR4/MD-2 in other species.

### Horse MD-2 does not fully abolish diC14-amidine signalling activity, in contrast to horse TLR4, in inter-species assays

To determine whether MD-2 or TLR4, or both, confer the observed species-specific differences in signalling, we conducted a series of MD-2/TLR4 swapping experiments (Fig. 1c). Cat TLR4/MD2, like human TLR4/MD2, was efficiently activated by diC14-amidine and comparative analysis between cat and human TLR4/MD2 was not likely to provide further information on how diC14-amidine interacts with this receptor complex. We focused therefore on human and horse comparisons. Whilst human TLR4 and MD-2 are fully activated by diC14-amidine nanoliposomes, and the equine TLR4 complex (eTLR4 + eMD-2) or the complex eTLR4 + hMD-2 are not, we still observed a partial activation of NF-κB occurring by stimulating with diC14-amidine the combination of hTLR4 and eMD2 (Fig. 1c). In contrast, EC-LPS was able to fully activate all combinations of human and horse TLR4 and MD-2 (see Fig. S3).

The importance of TLR4 over MD-2 suggests a different mode of TLR4 activation by diC14-amidine as compared to the more classical LPS derivatives. By comparison, similar experiments made with lipid IVa (agonist in horse and mouse but antagonist in human) showed that this ligand required both horse TLR4 and horse MD-2 or both mouse TLR4 and MD-2 to be active [16, 19] (no activation was found with combination of hTLR4/eMD-2 or eTLR4/hMD-2) which was further confirmed by the crystal structures of mouse TLR4/MD-2/lipid IVa and human MD-2/lipid IVa [4, 17]. Interestingly, another human TLR4 activator has been reported to mediate TLR4 signalling irrespectively of the origin of the transfected MD-2 co-receptor: nickel ions [30]. Schmidt and colleagues [30] proposed that nickel ions activate NF-κB through binding of species-specific histidine residues in TLR4, triggering the formation of a TLR4/MD-2:TLR4*/MD-2* dimer that structurally resembles the one induced by LPS [30]. Although MD-2 is required for TLR4 dimerization, it does not participate in nickel binding explaining why species dependency of the agonist activity of nickel ions is solely dependent on human TLR4 [31].

### Antagonism of diC14-amidine activation of TLR4

**Fig. 2:**
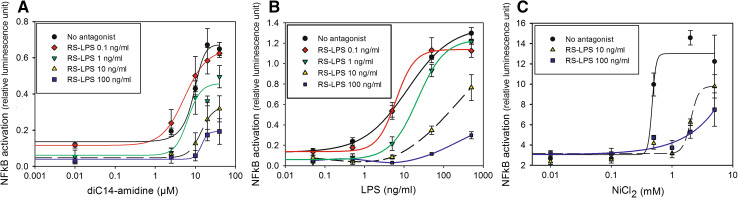
Antagonist effect of RS-LPS on diC14-amidine, LPS and nickel TLR4 agonist activities. HEK 293 cells were transfected with plasmids encoding human CD14, MD-2, and TLR4, together with reporter plasmids. Two days after transfection, cells were pretreated with the indicated amount of RS-LPS for 1 h, and then washed twice. Cells were then stimulated with diC14-amidine (**a**), LPS (**b**) or NiCl_2_ (**c**), for 6 h, and luciferase was quantified in cell lysates. Means are expressed ± standard deviation with *n* = 3. Representative of at least 2 independent experiments

To determine whether the recognition interface of diC14-amidine is different from the known LPS-binding site, we compared the effect of a TLR4 antagonist, *Rhodobacter sphaeroides LPS* (RS-LPS), on the TLR4 agonist activity of diC14-amidine and *E. coli* LPS (Fig. 2). RS-LPS (Fig. S1) is a potent antagonist for human TLR4, interacting with TLR4/MD-2 by inserting its lipid tails into MD-2’s binding pocket [13].

Figure 2 shows the dose–response curves for EC-LPS (*B*) and diC14-amidine (*A*) upon pretreatment with increasing concentrations of RS-LPS. As expected, RS-LPS and EC-LPS compete for the same binding site, so the potency of the response to LPS was reduced after pretreatment with RS-LPS, but with no alteration of the maximal response reached at high concentrations of EC-LPS (i.e. showing a parallel rightward shift of agonist dose–response curves). In contrast, RS-LPS decreases the potency and magnitude of the maximum response of diC14-amidine (RS-LPS effect cannot be negated, no matter how much diC14-amidine is present). This non-competitive antagonism of RS-LPS on diC14-amidine’s activity in contrast to the competitive antagonism seen for EC-LPS suggests that diC14-amidine binds at a different site on the TLR4/MD-2 complex. The same behaviour was found for nickel ions (Fig. 2c), underlining the similarities between these two TLR4 activators.

**Fig. 3:**
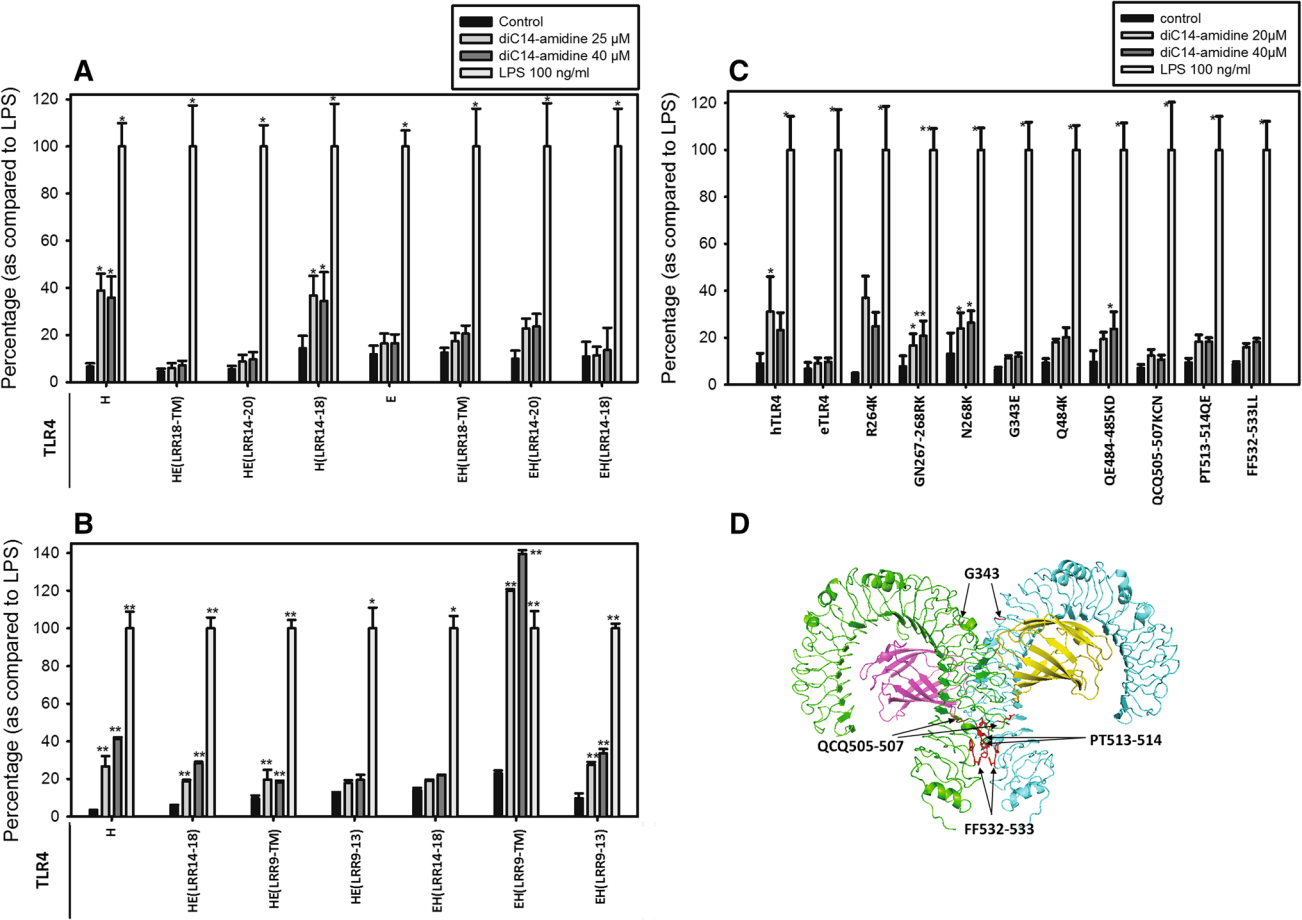
TLR4 leucine-rich repeats LRR 9–13 and 18–20 are important for the activity of diC14-amidine. **a**, **b**, **c** HEK 293 cells were transfected with plasmids encoding human CD14, MD-2, and chimeric/mutant TLR4, together with reporter plasmids, then stimulated 48 h later with diC14-amidine for 6 h before quantification of NF-κB activation. Luciferase was then quantified in cell lysates. Data are represented as fold induction as compared to non-stimulated condition (control) for each species. Means are expressed ± standard deviation with *n* = 3–15. Representative of at least 2 independent experiments. *p < 0.05, **p < 0.01 as compared to control (ANOVA). **d** Localisation of the important residues for diC14-amidine’s agonist activity based on the known structure of TLR4/MD-2/LPS [3]

To identify the regions of TLR4 involved in the diC14-amidine agonist activity, we tested the ability of human/horse chimeras, in which regions of TLR4 from one species are exchanged with the corresponding ones from the other species, to be activated by diC14-amidine (Fig. 3a, b). This approach was previously used to identify residues in TLR4 and MD-2 that are important for the recognition of lipid IVa [14, 16, 19, 20] or RS-LPS [13] as TLR4 agonists and which were further confirmed by the crystal structures of human TLR4/MD2/EC-LPS, mouse TLR4/MD-2/lipid IVa and human MD-2/lipid IVa [3, 4, 17]. We constructed several chimeras first based on the regions known to be important in the case of lipid IVa or RS-LPS. Indeed, for these ligands, the LRR 14–18 region is critical for their agonist activity in horse TLR4 and corresponds to the dimerization interface in the C-terminal domain interacting with one lipid chain of LPS [3]. A second region was also explored, corresponding to the primary binding interface (i.e. before ligand binding) between TLR4 and MD-2 located in the concave surface (LRR 9–13) of TLR4 [3] which is also known to interact with LPS derivative headgroups.

The chimeras HE (LRR 14–18) (human TLR4 with the equine LRR 14–18 insert), EH (LRR18-TM) (equine TLR4 with the human LRR 18-TM insert) and EH (14–20) (equine TLR4 with the human LRR 14–20 insert) are activated by diC14-amidine (Fig. 3a). Since these chimeras have only the human region LRR 18–20 in common (Fig. S4), this result suggests that residues 484–535 are critical for diC14-amidine recognition as an agonist. Another set of chimeras were also activated by diC14-amidine: HE (LRR 14–18) (already mentioned here above), EH (LRR9-TM) (equine TLR4 with the human LRR 9-TM insert) and EH (LRR9-13) (equine TLR4 with the human LRR 9–13 insert) (Fig. 3b), suggesting that a second region, corresponding to LRR 9–13 (see Fig. S4) (i.e. residues 252–370) is also involved in TLR4 agonist activity of diC14-amidine.

### Species-specific point mutagenesis identifies in TLR4 critical residues for the recognition of diC14-amidine

The comparison of the human and equine sequences in the LRR 9–13 and 18–20 regions (Fig. S5) reveals that only 20 amino acids are different between horse and human in the LRR 18–20 region and 44 residues in the LRR 9–13 region. To identify the residues in human TLR4 important for diC14-amidine interaction, we produced several point mutations of human TLR4 whereby one or two residues were replaced by their equine homologues: R264K, GN267-8RK, N268K, G343E, Q484K, QE484-5KD, QCQ505-7KCN, PT513-4QE and FF532-3LL using site-directed mutagenesis, and tested their activity in response to diC14-amidine (Fig. 3c). Most mutants responded similarly to wild-type human TLR4 to diC14-amidine, while mutants FF532-533LL and PT513-514QE showed a decreased activity in response to diC14-amidine and mutants G343E and QCQ505-7KCN were not activated by diC14-amidine, similar to equine TLR4. Interestingly, amino acids G343, Q505, Q507 and F533 do not interact with LPS in the crystal structure of TLR4/MD-2/LPS [3] but are important in TLR4-TLR4* interface interaction. The residues corresponding to Q507 and Q344 in horse (N508 and G345) are unique to this species (Fig. S5) and may therefore explain the difference of diC14-amidine’s TLR4 agonist activity for horse as compared to other species.

It is striking that the mutated residues that lead to a loss (or a decrease) of the TLR4 agonist activity of diC14-amidine are located in the TLR4-TLR4* dimerization interface (see Fig. 3d), while TLR4 residues involved in the interaction with the headgroup or the lipid chains of LPS [3] do not influence diC14-amidine’s agonist activity. This strongly suggests that diC14-amidine interacts with TLR4 via a mechanism different to that previously proposed for LPS and its derivatives [3, 17]. Therefore, although the lipidic nature of diC14-amidine suggested that it was likely to interact with MD-2 and possess the so-called MD-2-related lipid-recognition domain [32] to activate the TLR4 pathway, our pharmacological analysis and mutagenesis data now suggests that this is unlikely.

### TLR4 hydrophobic crevices are potential binding sites for diC14-amidine

**Fig. 4:**
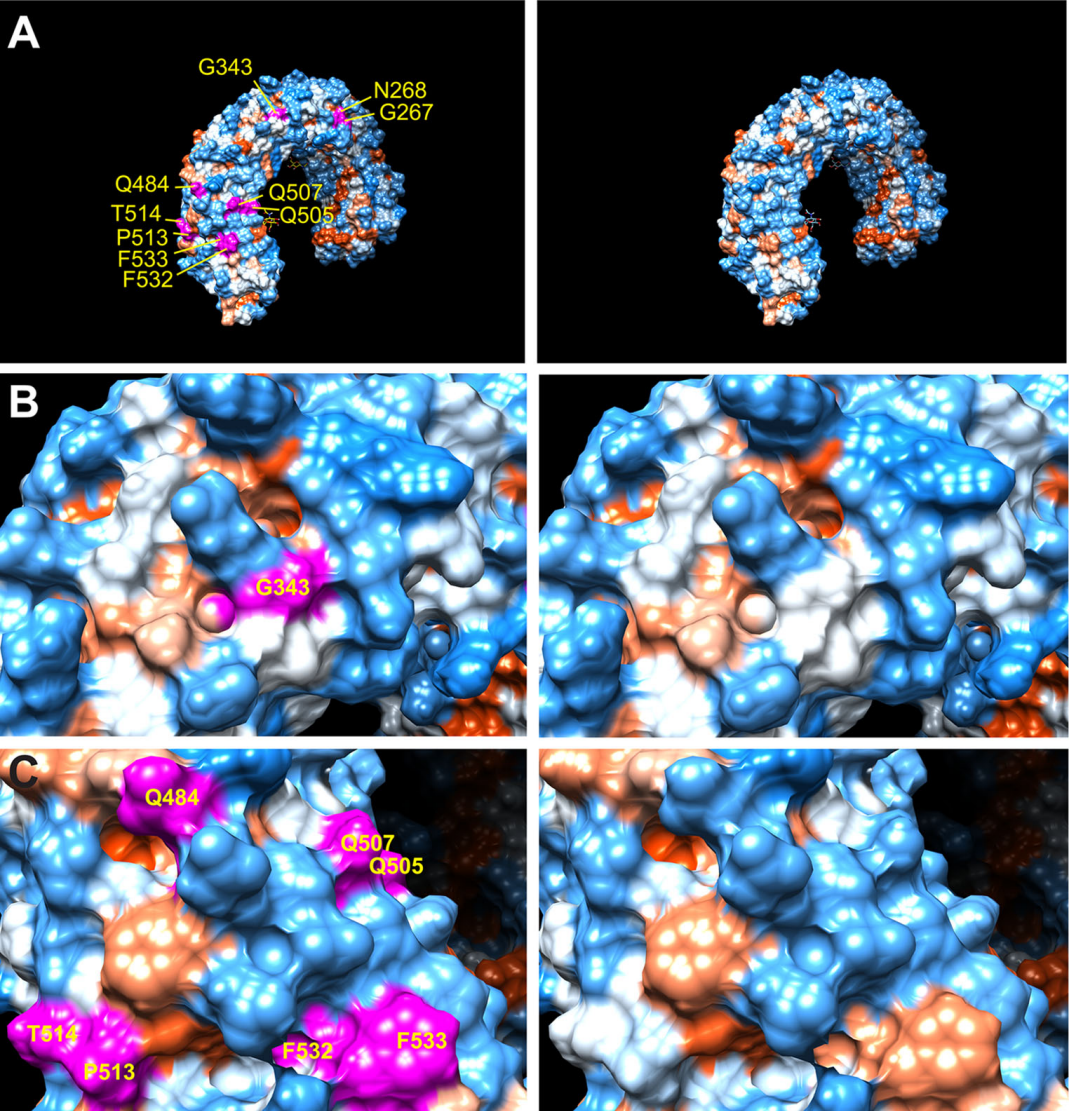
Hydrophobic crevices on the surface of TLR4’s ectodomain. **a** Molecular surface of the TLR4 ectodomain coloured according to its hydrophobicity (*orange* hydrophobic, *blue* hydrophilic). Species-specific residues that have been targeted by point mutagenesis are highlighted in *magenta* in the *left panels*. **b**, **c** Close-up views illustrate the residues’ proximity to hydrophobic crevices, potentially involved in diC14-amidine binding

**Fig. 5:**
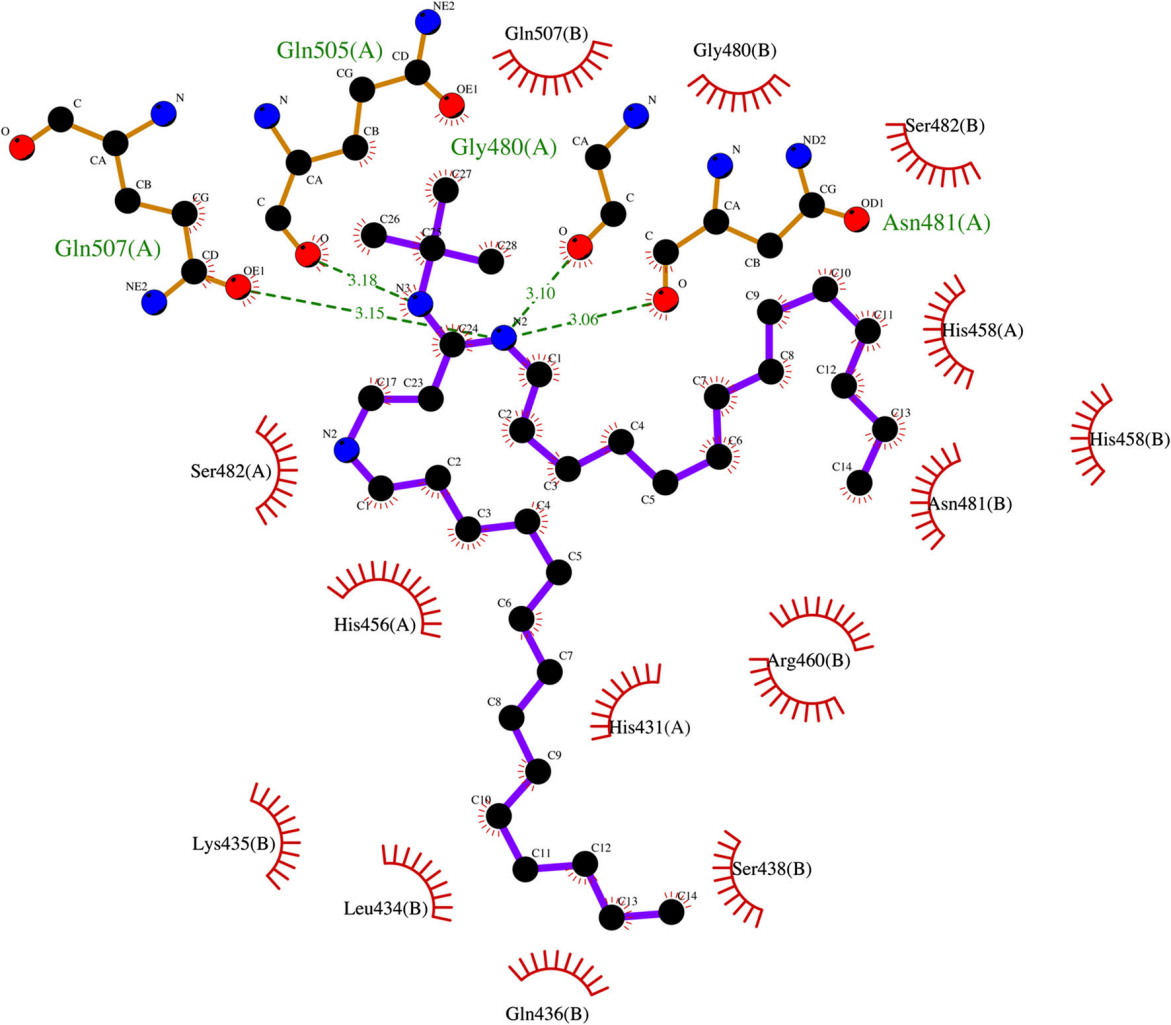
Potential docking of diC14-amidine within the TLR4 dimer interface. Docking pause at −5.2 kcal/mol involving hydrogen bonds with the side chain of Gln 507 and the main chain carbonyl groups of Gly 480, Asn 481, and Gln 505, as well as a number of hydrophobic contacts at the TLR4 dimer interface. Figure generated by LigPlot [29]

TLR4 possesses hydrophobic crevices that are spread all over its leucine and cysteine-rich regions with volumes up to 335 Å^3^ according to CastP server calculations [33] (Fig. 4). We postulate, therefore, that diC14-amidine might be able to bind TLR4 through interaction with its hydrophobic crevices, with the possibility of binding several diC14-amidine molecules to several hydrophobic crevices throughout the TLR4-TLR4* interface. However, none of the hydrophobic grooves in TLR4 are as deep as those found in TLR2 which help to form TLR2-TLR1 and TLR2-TLR6 heterodimers in the presence of bacterial tri- and di-acylated lipopeptides (BLPs), respectively [34, 35]. BLPs are indispensable to hold these ectodomains together via hydrophilic and hydrophobic interactions. While two ester-bound acyl chains are inserted into a pocket in TLR2, the amide-bound lipid of triacylated BLPs is fitted into a hydrophobic channel in TLR1. The latter is blocked off by Phe residues in TLR6 explaining the ligand specificity of the system [34, 35]. To clarify the mode of action of diC14-amidine, we generated docking models for this molecule in complex with the TLR4 ectodomain and the dimeric TLR4/MD-2 complex [3]. DiC14-amidine could be docked in the vicinity of LRR19 residues Q505 and Q507 [at −5.2 kcal/mol and with an apparent K_i_ value in the micromolar range (150 µM)]. This putative binding site is located at the TLR4-TLR4* interface with the headgroup wedged between Q505 and Q507 and both myristate chains at the ascending flanks of TLR4 LRR 16–19 (Fig. 5). The latter Gln residue is conserved in human, mouse and cat and replaced by an Asn in horse TLR4. The shorter side chain in horse might explain the weaker activity of diC14-amidine in horse compared to other mammalian species. Therefore, our docking model proposes a potential binding site for diC14-amidine molecules in the proximity of the hydrophobic crevices found to be important in this study.

## Discussion

Here, we show how the cationic lipid diC14-amidine, a molecule initially designed for use as a lipid-based nanocarrier in gene therapy, is immunostimulatory. Our data suggest that diC14-amidine interacts directly with TLR4 and induces its dimerization via cross-linking two receptor chains. The subsequent cell signalling mechanism is analogous to the one that was proposed for TLR4 activation by nickel ions [30]. As already mentioned, it was previously proposed that nickel and cobalt ions trigger MD-2 dependent TLR4 dimerization and activation through chelation of species-specific histidine residues (H431, H456 and H458 on both TLR4 and TLR4*) located at the dimerization interface of human TLR4-TLR4 [30]. Oblak et al. [31] recently confirmed that nickel-binding site is completely independent of the endotoxin-binding site but proposed that MD-2 nevertheless contributes to the interaction by providing supporting hydrophobic interactions with TLR4 which stabilize the TLR4/MD-2/Ni2+ complex in a proper conformation for cellular activation.

Cross-linking of TLR4 ectodomains, resulting in their activation, may also occur following incubation of cells with antibodies directed against TLR4 [36, 37]. This implies that different anchoring points can lead to dimerization and foresees other possible ways of TLR4 activation that have not been explored until now. It is likely that, in the future, other microbial or endogenous TLR4 stimulators (like amino acid-containing lipid present in many Gram-negative bacteria [38, 39], but also gangliosides [40] or ceramides [41, 42], which all share common structural features with diC14-amidine) will be demonstrated to be able to activate TLR4 through interaction with the TLR4 dimerization interface, expanding the possible ligands of TLR4 to non-MD-2-binding lipids. Finally, this new recognition interface might also be involved in the recognition of nanoparticles by the innate immune system. Indeed, diC14-amidine liposomes are not the only example of lipid-based nanoparticles, or more generally of engineered nanoparticles, that activate Toll-like receptor 4 [26, 43–46]. Our work can therefore contribute to a deeper knowledge of the effects of engineered nanoparticles on the immune system, a necessary step for their safer use in nanomedicine and for an improved therapeutic efficacy.

In addition, our data also demonstrate that diC14-amidine does not require CD14 to activate MyD88-dependent pathways. Similar CD14-independent behaviour has been reported for two synthetic lipid A derivatives: MPL (Monophosphoryl Lipid A) and CRX-527 and for the rough form of LPS (LPS lacking the full-length O-antigen chains—see Fig. S1) [47–50]. CD14 ligand carrier role has been established beyond doubt and is involved in the presentation of LPS to TLR4/MD-2 for initiating the MyD88-dependent pathway [51]. The lack of the long polysaccharide chains in these LPS derivatives probably allows their better incorporation into and higher mobility in the mammalian cell membrane, providing a better access to protein receptors [48]. Therefore, the ability of diC14-amidine, with its small hydrophilic headgroup, to be inserted into cell membranes after fusion [52, 53] offers a straightforward explanation for why this ligand does not require CD14 for inducing the MyD88-dependent pathway.

Finally, while it was generally accepted that CD14 was required for the LPS-induced endocytosis of TLR4 [54] which is considered necessary to enable the activation of the TRIF-dependent pathway [51, 55], our results demonstrate that CD14 is also not required to trigger the TRIF-dependent pathway induced by diC14-amidine, in contrast to stimulation by rough LPS, MPL and CRX-527 [47–50]. Such behaviour has been described for LPS-coated latex beads [54] or LPS-formulated liposomes [56], which showed enhanced LPS endocytosis in the absence of CD14 (as compared to free LPS) thus confirming the importance of endocytosis for the TRIF-dependent signalling pathway [54, 55]. However, those LPS formulations were found unable to activate the My88-dependent signalling pathway from inside the endosomes [56]. The uniqueness of diC14-amidine, as compared to other known TLR4 ligands, to activate both signalling pathways in the absence of CD14, may therefore be related to the fact that diC14-amidine liposomes enter the cells via both endocytosis and fusion processes [53, 57].

In conclusion, here we show that the TLR4 agonist activity of the cationic lipid nanocarrier diC14-amidine is primarily dependent on its interaction with TLR4 by a mechanism likely similar to that proposed for nickel and cobalt ions. Important residues located at the N- and C-terminal edges of the TLR4/TLR4* dimerization interface are distinct from those reported for LPS binding and explain why two molecules as structurally different as diC14-amidine and LPS are both TLR4 activators. This may represent a new lead in developing compounds targeting these interactions in TLR4 without affecting the other functionalities of the receptor, in particular the recognition of conventional ligands.

### Electronic supplementary material

Below is the link to the electronic supplementary material.

***Figure S1***: ***E. coli LPS (EC-LPS), Rhodobacter sphaeroides Lipopolysaccharide (RS-LPS) and diC14-amidine structures.*** In their general architecture, LPS molecules consist of a hydrophobic part named ‘lipid A’ covalently attached to a polysaccharide region made of a rather well-conserved ‘core’ oligosaccharide backbone, and an highly variable outer chain (‘O-antigen’) consisting of a complex polymer of oligosaccharides (TIFF 1929 kb)

***Figure S2***: ***Effect of CD14 neutralizing antibodies on the MyD88-dependent and TRIF-dependent cell response of primed THP1 cells after stimulation with EC-LPS or diC14-amidine.*** After priming for 24 hours with PMA followed by 4 hours in complete medium the cells to be treated with the control antibody (Control-IgA2) or blocking antibody against CD14 (Anti-hCD14-IgA) were incubated with 20 µg/ mL antibody in RPMI for the control and for the cells to be stimulated with diC14-amidine and with the same concentration of antibodies in complete medium in the case of subsequent EC-LPS stimulation. After 1 hour of incubation concentrated stimulants were added to the cells to reach final stimulant concentrations of 10 ng/ mL for EC-LPS and 20 µM for diC14-amidine. After 4 hours of stimulation the supernatant was recovered and the secretion of hTNF-α was quantified by ELISA. n = 3, means ± s.d.; ND = Not detected, i.e. at the minimum reporting level (TIFF 4474 kb)

***Figure S3***: ***DiC14-amidine activates partially human TLR4+equine MD-2 complex.*** HEK 293 cells were transfected with plasmids encoding human TLR4, MD-2 and CD14 from human (H) or horse (E), together with firefly luciferase reporter plasmid dependent of NF-κB activation. Two days after transfection cells were stimulated for 6h with diC14-amidine 10 µM or LPS 100 ng/ml. Luciferase was then quantified in cell lysates. Data are represented as fold induction as compared to non-stimulated control for each condition. Means are expressed +/- standard deviation with n = 3. Figure representative of at least 2 independent experiments. **: p<0.01 as compared to control (ANOVA). Comparison between groups were done by two-way analysis ANOVA. S: significant difference, NS: non-significant (TIFF 8128 kb)

***Figure S4***: ***Representation of the different TLR4 chimeras used in this work.*** The overall structure of TLR4 is represented on the top (***A***). (***B***) Chimeras were constructed by exchanging specific region in the TLR4 coding plasmid for one species by its corresponding region from the other species using overlap extension PCR. (***C***) The two sets of chimeras activated by diC14-amidine (see *Fig. 3*) define two regions in human TLR4 important for diC14-amidine’s agonist activity (TIFF 910 kb)

***Figure S5:*** ***Multiple sequence alignment (MSA) of the equine, human, murine and feline TLR4 sequences in the two regions identified by the chimera assays using Clustal Omega (https://www.ebi.ac.uk/Tools/msa/clustalo/).*** NCBI Reference Sequences NP_001093239, NP_612564.1, NP_067272.1 and NP_001009223. The last line of a MSA block labels the homology relationship (an “*” indicates a fully conserved residue, a “:” a conservation between groups of strongly similar properties, a “.” indicates a conservation between groups of weakly similar properties, while blank space marks missing homology). Colours refer to physicochemical properties of amino acids. Red indicates small + hydrophobic residues (AVFPMILW), Blue: acidic (DE), Magenta: basic (RK), Green: Hydroxyl + sulfhydryl + amine (STYHCNGQ) (TIFF 62 kb)
