# Supplementary figures and images for: Critical residues involved in Toll-like receptor 4 activation by cationic lipid nanocarriers are not located at the lipopolysaccharide-binding interface
Source: Cell Mol Life Sci. 2015 May 9;72(20):3971–82. doi: 10.1007/s00018-015-1915-1 (PMC4575701; doi:10.1007/s00018-015-1915-1)

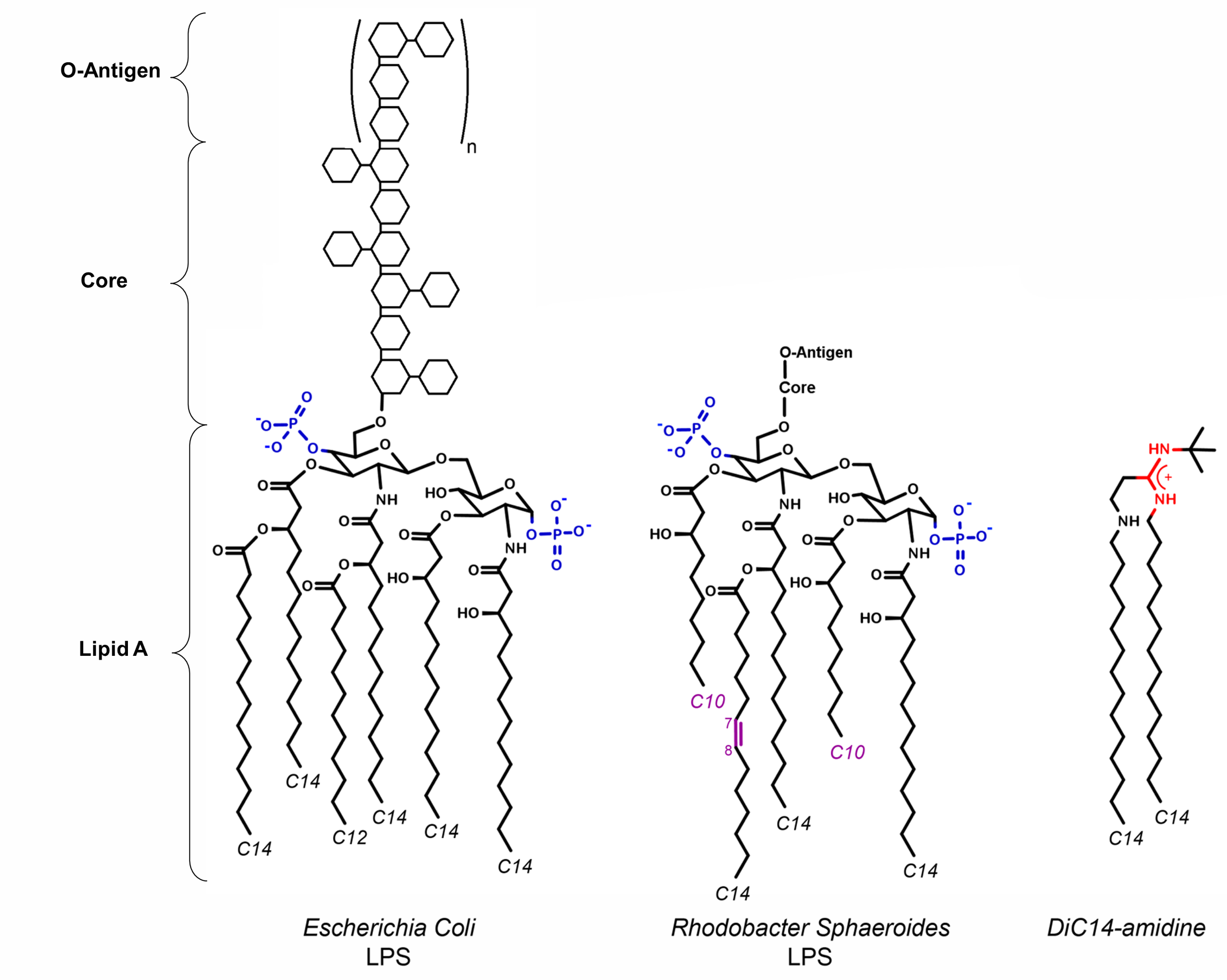

Supplement: Supplementary file 1 — Figure S1: E. coli LPS (EC-LPS), Rhodobacter sphaeroides Lipopolysaccharide (RS-LPS) and diC14-amidine structures. In their general architecture, LPS molecules consist of a hydrophobic part named ‘lipid A’ covalently attached to a polysaccharide region made of a rather well-conserved ‘core’ oligosaccharide backbone, and an highly variable outer chain (‘O-antigen’) consisting of a complex polymer of oligosaccharides (TIFF 1929 kb) [file 18_2015_1915_MOESM1_ESM.tif]

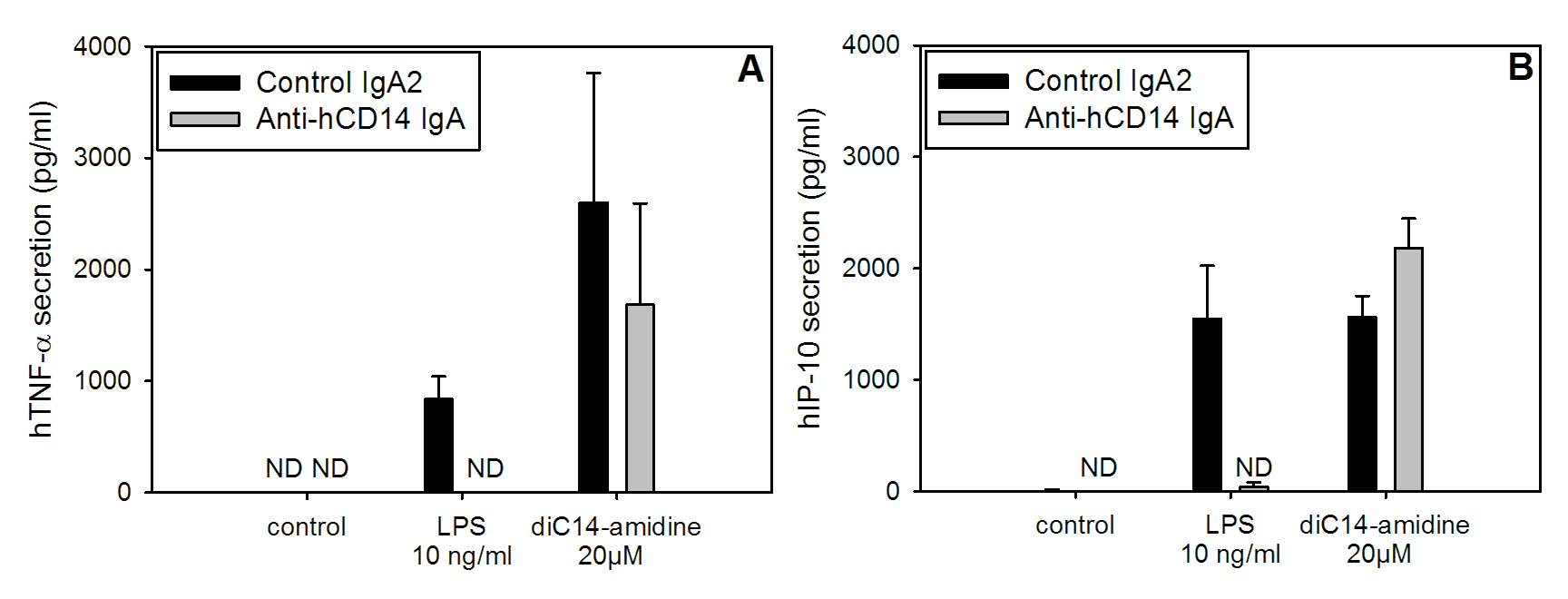

Supplement: Supplementary file 2 — Figure S2: Effect of CD14 neutralizing antibodies on the MyD88-dependent and TRIF-dependent cell response of primed THP1 cells after stimulation with EC-LPS or diC14-amidine. After priming for 24 hours with PMA followed by 4 hours in complete medium the cells to be treated with the control antibody (Control-IgA2) or blocking antibody against CD14 (Anti-hCD14-IgA) were incubated with 20 µg/ mL antibody in RPMI for the control and for the cells to be stimulated with diC14-amidine and with the same concentration of antibodies in complete medium in the case of subsequent EC-LPS stimulation. After 1 hour of incubation concentrated stimulants were added to the cells to reach final stimulant concentrations of 10 ng/ mL for EC-LPS and 20 µM for diC14-amidine. After 4 hours of stimulation the supernatant was recovered and the secretion of hTNF-α was quantified by ELISA. n = 3, means ± s.d.; ND = Not detected, i.e. at the minimum reporting level (TIFF 4474 kb) [file 18_2015_1915_MOESM2_ESM.tif]

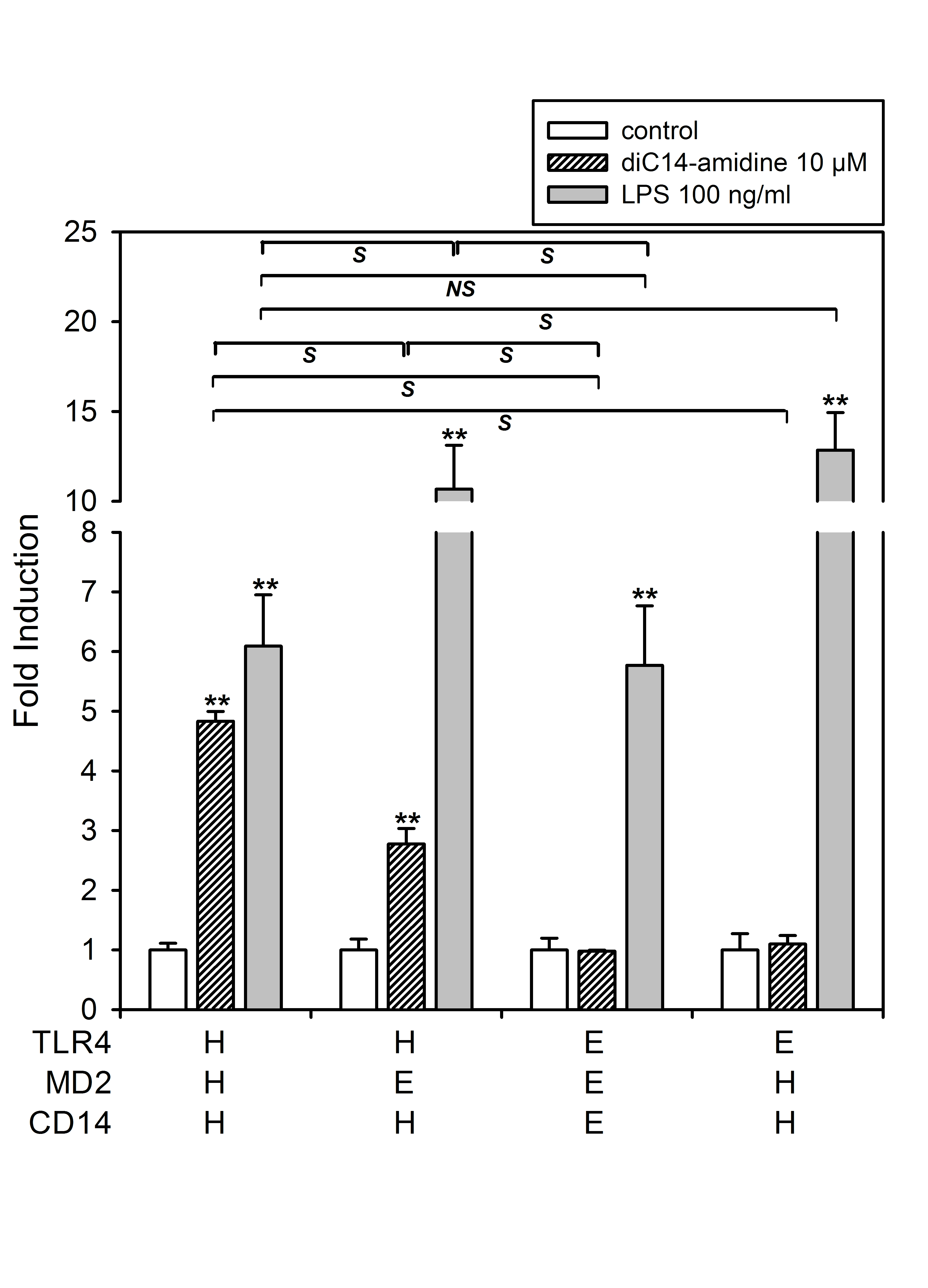

Supplement: Supplementary file 3 — Figure S3: DiC14-amidine activates partially human TLR4+equine MD-2 complex. HEK 293 cells were transfected with plasmids encoding human TLR4, MD-2 and CD14 from human (H) or horse (E), together with firefly luciferase reporter plasmid dependent of NF-κB activation. Two days after transfection cells were stimulated for 6h with diC14-amidine 10 µM or LPS 100 ng/ml. Luciferase was then quantified in cell lysates. Data are represented as fold induction as compared to non-stimulated control for each condition. Means are expressed +/- standard deviation with n = 3. Figure representative of at least 2 independent experiments. **: p<0.01 as compared to control (ANOVA). Comparison between groups were done by two-way analysis ANOVA. S: significant difference, NS: non-significant (TIFF 8128 kb) [file 18_2015_1915_MOESM3_ESM.tif]

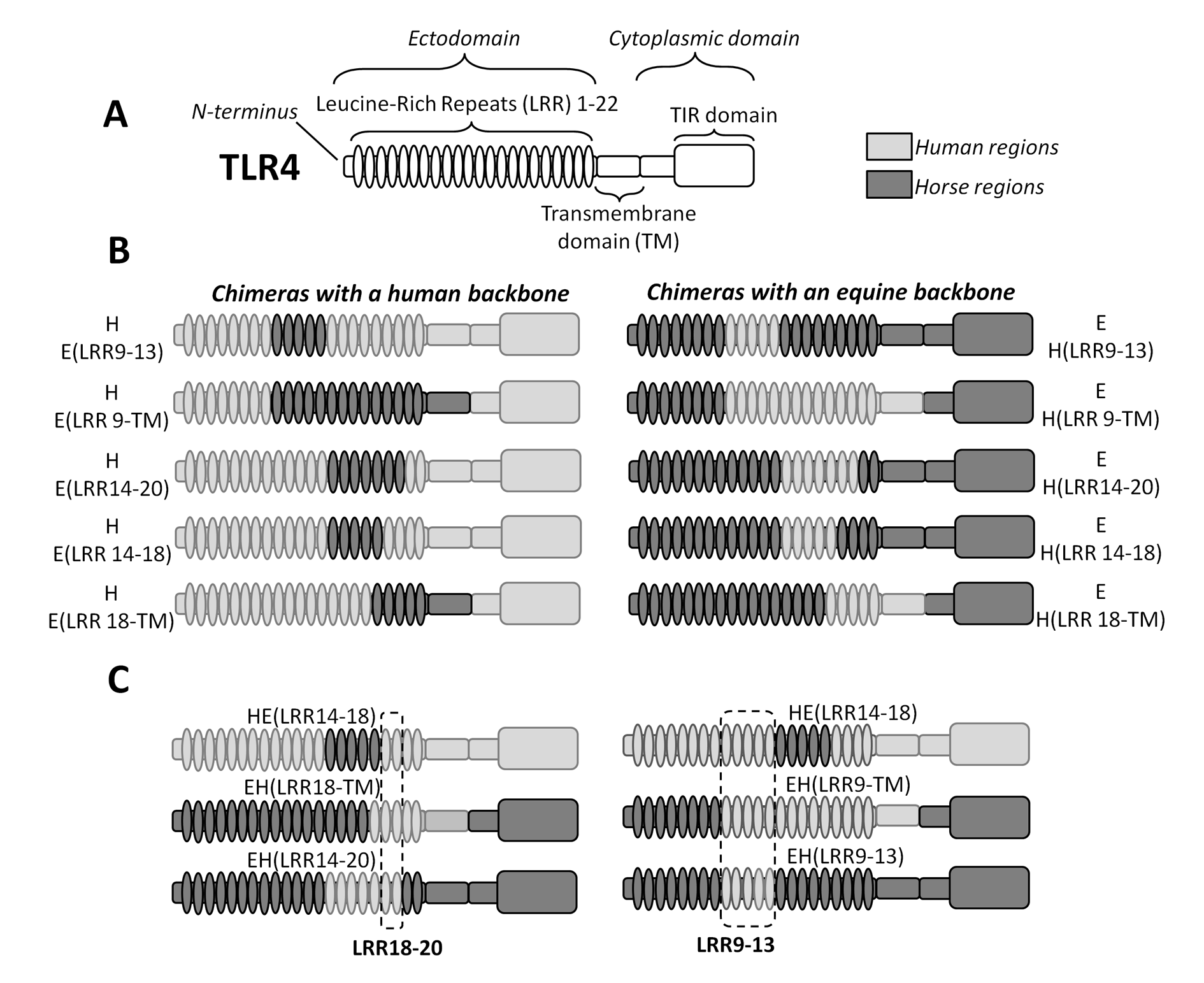

Supplement: Supplementary file 4 — Figure S4: Representation of the different TLR4 chimeras used in this work. The overall structure of TLR4 is represented on the top (A). (B) Chimeras were constructed by exchanging specific region in the TLR4 coding plasmid for one species by its corresponding region from the other species using overlap extension PCR. (C) The two sets of chimeras activated by diC14-amidine (see Fig. 3) define two regions in human TLR4 important for diC14-amidine’s agonist activity (TIFF 910 kb) [file 18_2015_1915_MOESM4_ESM.tif]

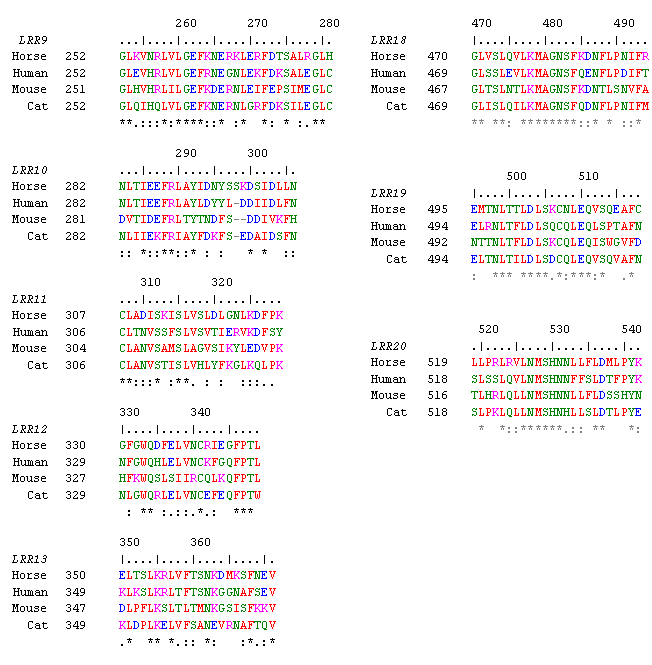

Supplement: Supplementary file 5 — Figure S5: Multiple sequence alignment (MSA) of the equine, human, murine and feline TLR4 sequences in the two regions identified by the chimera assays using Clustal Omega (https://www.ebi.ac.uk/Tools/msa/clustalo/). NCBI Reference Sequences NP_001093239, NP_612564.1, NP_067272.1 and NP_001009223. The last line of a MSA block labels the homology relationship (an “*” indicates a fully conserved residue, a “:” a conservation between groups of strongly similar properties, a “.” indicates a conservation between groups of weakly similar properties, while blank space marks missing homology). Colours refer to physicochemical properties of amino acids. Red indicates small + hydrophobic residues (AVFPMILW), Blue: acidic (DE), Magenta: basic (RK), Green: Hydroxyl + sulfhydryl + amine (STYHCNGQ) (TIFF 62 kb) [file 18_2015_1915_MOESM5_ESM.tif]
